# Left Ventricular Ejection Fraction Association with Acute Ischemic Stroke Outcomes in Patients Undergoing Thrombolysis

**DOI:** 10.3390/jcdd10060231

**Published:** 2023-05-25

**Authors:** Ryan C. H. Chee, Norman H. Lin, Jamie S. Y. Ho, Aloysius S. T. Leow, Tony Y. W. Li, Edward C. Y. Lee, Mark Y. Chan, William K. F. Kong, Tiong-Cheng Yeo, Ping Chai, James W. L. Yip, Kian-Keong Poh, Vijay K. Sharma, Leonard L. L. Yeo, Benjamin Y. Q. Tan, Ching-Hui Sia

**Affiliations:** 1 Department of Medicine, Yong Loo Lin School of Medicine, National University of Singapore, Singapore 117597, Singapore; ryancch1@gmail.com (R.C.H.C.); leowaloysius@hotmail.com (A.S.T.L.); mark.chan@nus.edu.sg (M.Y.C.); william_kong@nuhs.edu.sg (W.K.F.K.); tiong_cheng_yeo@nuhs.edu.sg (T.-C.Y.); ping_chai@nuhs.edu.sg (P.C.); james_yip@nuhs.edu.sg (J.W.L.Y.); mdcpkk@nus.edu.sg (K.-K.P.); vijay_kumar_sharma@nuhs.edu.sg (V.K.S.); leonardyeoll@gmail.com (L.L.L.Y.); benjaminyqtan@gmail.com (B.Y.Q.T.); 2 Department of Cardiology, National University Heart Centre Singapore, Singapore 119074, Singapore; normanhylin@gmail.com (N.H.L.); sinyingh@gmail.com (J.S.Y.H.); tonyli92@gmail.com (T.Y.W.L.); edward.lee.cy@hotmail.com (E.C.Y.L.); 3 Division of Neurology, Department of Medicine, National University Hospital, Singapore 119074, Singapore

**Keywords:** left ventricular systolic dysfunction, IV thrombolysis, acute ischemic stroke, functional and clinical outcomes

## Abstract

(1) Background: Little is known about how left ventricular systolic dysfunction (LVSD) affects functional and clinical outcomes in acute ischemic stroke (AIS) patients undergoing thrombolysis; (2) Methods: A retrospective observational study conducted between 2006 and 2018 included 937 consecutive AIS patients undergoing thrombolysis. LVSD was defined as left ventricular ejection fraction (LVEF) < 50%. Univariate and multivariate binary logistic regression analysis was performed for demographic characteristics. Ordinal shift regression was used for functional modified Rankin Scale (mRS) outcome at 3 months. Survival analysis of mortality, heart failure (HF) admission, myocardial infarction (MI) and stroke/transient ischemic attack (TIA) was evaluated with a Cox-proportional hazards model; (3) Results: LVSD patients in comparison with LVEF ≥ 50% patients accounted for 190 and 747 patients, respectively. LVSD patients had more comorbidities including diabetes mellitus (100 (52.6%) vs. 280 (37.5%), *p* < 0.001), atrial fibrillation (69 (36.3%) vs. 212 (28.4%), *p* = 0.033), ischemic heart disease (130 (68.4%) vs. 145 (19.4%), *p* < 0.001) and HF (150 (78.9%) vs. 46 (6.2%), *p* < 0.001). LVSD was associated with worse functional mRS outcomes at 3 months (adjusted OR 1.41, 95% CI 1.03–1.92, *p* = 0.030). Survival analysis identified LVSD to significantly predict all-cause mortality (adjusted HR [aHR] 3.38, 95% CI 1.74–6.54, *p* < 0.001), subsequent HF admission (aHR 4.23, 95% CI 2.17–8.26, *p* < 0.001) and MI (aHR 2.49, 95% CI 1.44–4.32, *p* = 0.001). LVSD did not predict recurrent stroke/TIA (aHR 1.15, 95% CI 0.77–1.72, *p* = 0.496); (4) Conclusions: LVSD in AIS patients undergoing thrombolysis was associated with increased all-cause mortality, subsequent HF admission, subsequent MI and poorer functional outcomes, highlighting a need to optimize LVEF.

## 1. Introduction

Stroke is the second leading cause of death and third leading cause of disability worldwide [1]. Intravenous (IV) thrombolysis remains a keystone in the treatment of acute ischemic stroke (AIS) and is recommended in the guidelines for administration within 4.5 h of ischemic stroke onset [2].

Heart disease is an important risk factor for AIS [3]. Left ventricular systolic dysfunction (LVSD) was associated with increased risk of ischemic stroke [4] in the SAVE (Survival and Ventricular Enlargement) trial, highlighting an 18% increase in stroke risk for every 5% decrease in left ventricular ejection fraction (LVEF) [5]. Possible underlying mechanisms for the relationship between heart disease and AIS may be due to LVSD causing a hypoperfusion state [6] with decreased global cerebral blood flow [7], as well as reduced cerebrovascular reactivity [8] and, hence, a lack of compensatory cerebrovascular reserve, which leads to a higher risk of AIS with an ischemic insult. Other postulated mechanisms are pro-thrombotic and pro-inflammatory states and hypoxia with LVSD [9,10,11].

Studies have highlighted a poorer prognosis in AIS patients with reduced LVEF [12,13] and a higher stroke severity at admission [6] in AIS patients with reduced LVEF. This translates into increased mortality [13,14] as well as poor functional outcomes [15] in AIS patients undergoing revascularization therapy with IV thrombolysis or endovascular thrombectomy (ET) [16].

However, despite the dearth of information available on the impact of LVSD on patients diagnosed with AIS, there is still a lack of data on the association between LVSD and IV thrombolysis. Furthermore, many studies include treatment options of both ET and IV thrombolysis in AIS patients, contributing to the heterogeneity of the data available, and, hence, resulting in an unclear impact of LVSD on AIS outcomes specifically in AIS patients undergoing IV thrombolysis. Therefore, we sought to investigate whether LVSD results in worse functional and clinical outcomes for AIS patients undergoing IV thrombolysis.

## 2. Materials and Methods

### 2.1. Study Design and Patient Demographics

We included consecutive AIS patients treated with IV thrombolysis between January 2006 and December 2018 at our tertiary academic center. Our tertiary academic center is a specialized stroke center with an IV thrombolytic therapy program and endovascular treatment for AIS. A multidisciplinary team comprising neurologists is involved and dedicated to stroke care for patients.

Patients with a suspected stroke are first escalated through the institution’s emergency physicians via the stroke protocol. Immediate assessments include a non-contrasted Computed Tomography (CT) brain scan and CT angiography performed in all patients with suspected stroke to assess the suitability for reperfusion therapy. Upon establishing reperfusion suitability in AIS patients, AIS patients within 4.5 h onset of stroke symptoms are administered 0.9 mg/kg of intravenous recombinant Tissue Plasminogen Activator with 10% of total dose as a bolus over the first 1 min and the remaining 90% of the total dose over 1 h, according to clinical guidelines. These patients are subsequently placed in a stroke unit for close monitoring and assessment of symptoms by the specialized stroke team.

A total of 937 AIS patients who had undergone IV thrombolysis and had a transthoracic echocardiogram (TTE) obtained within 6 months of the index AIS event. These patients were included in our study. All patient data were obtained via the Computerized Patient Support System in our hospital.

Stroke severity was assessed via the National Institutes of Health Stroke Scale (NIHSS) score [17] and modified Rankin Scale (mRS) score [18]. Other stroke characteristics included site of occlusion, stroke etiology via Trial of ORG 10172 in acute stroke treatment (TOAST) classification [19], time from stroke onset to needle, time from door-to-needle and recanalization success. Recanalization success was defined as the restoration of patency to the occluding site of a vessel and was further divided into complete recanalization, partial recanalization, no recanalization and no occlusion. With reference to the Arterial Occlusive Lesion score, complete recanalization was defined as uninterrupted blood flow with distal flow, partial recanalization was defined as continuous blood flow interruptions with narrowing of arterial lumen at the target artery with or without distal flow and absent recanalization was defined as blow flow that is completely interrupted [20].

Patient baseline characteristics and existing or newly diagnosed cardiovascular comorbidities were also collected including hypertension, dyslipidemia, diabetes mellitus, atrial fibrillation, ischemic heart disease and heart failure (HF). Hypertension, dyslipidemia and diabetes mellitus are defined according to the Singapore’s Ministry of Health Clinical Practice Guidelines [21]. Hypertension was defined as a systolic blood pressure of ≥140 mmHg or diastolic blood pressure of ≥90 mmHg for patients ≥ 18 years old who are not acutely ill and not consuming any antihypertensive medication [22]. Diabetes mellitus was defined as HbA1c > 7.0%, fasting plasma glucose ≥ 7.0 mmol/L, random plasma glucose level of ≥11.1 mmol/L or 2 h post challenge plasma glucose ≥ 11.1 mmol/L [23]. Dyslipidemia was defined as low-density lipoprotein cholesterol ≥ 3.4 mmol/L [24]. Information on cardiac-related interventions including percutaneous coronary intervention and coronary artery bypass graft was obtained from the electronic medical records.

### 2.2. Echocardiographic Data Acquisition and Analysis

TTE was used to assess LVEF and cardiac parameters. TTE images were recorded using commercially available ultrasound devices. TTE was performed with patients resting in the left lateral decubitus position. Electrocardiogram-triggered echocardiographic data were acquired and digitally stored in cine-loop format for offline analysis. LVEF was measured using the Simpson’s biplane method of discs according to international guidelines [25]. This was based on the tracing of the endocardial border in both the apical four-chamber and two-chamber views in end-systole and end-diastole obtained by a trained echocardiographer.

AIS patients were divided into 2 categories: patients with and without LVSD. LVSD was defined as LVEF < 50% [26]. An LVEF of 50% was used, as this value is still clinically used as per the American College of Cardiology guidelines [27] and this value aids in distinguishing between normal and dysfunctional LVEF as per the American Society of Echocardiography and the European Association of Cardiovascular Imaging [25].

### 2.3. Evaluation of Outcomes

The primary outcomes included functional independence at 3 months, while secondary outcomes evaluated included all-cause mortality, subsequent HF admission, subsequent myocardial infarction (MI) event, recurrent stroke/transient ischemic attack (TIA) event and symptomatic intracerebral hemorrhage (ICH). We defined functional independence as an mRS score of 0 to 2 at 3 months [28].

For all evaluated clinical outcomes, the date of the outcomes was noted. The underlying cause for mortality was noted down and further categorized into cardiac, noncardiac and unknown causes. Subsequent HF, MI and recurrent stroke/TIA event were defined as the next earliest occurrence of the respective event in patients post AIS with IV thrombolysis.

### 2.4. Statistical Analysis

We included variables that were clinically relevant for analysis. Normally distributed continuous variables were presented as mean ± standard deviation (SD), while categorical variables were presented as percentages. We used Pearson χ^2^ test (or Fisher exact test where applicable) for categorical variables and Student’s *t*-test for normally distributed continuous variables. A multivariate binary logistic regression model was constructed to identify independent predictors of LVSD. These findings were presented as adjusted odds ratios (aORs) with their corresponding 95% confidence interval (CI) and *p* value. Univariate analysis was first performed and variables with *p* values < 0.05 or deemed as clinically significant confounders were then included into the multivariable model. Ordinal shift regression analysis was used to evaluate functional independence based on the presence of LVSD. Cox proportional hazards analysis was performed for clinically relevant outcomes including mortality, subsequent episodes of MI, HF admission and stroke/TIA. These findings were presented as adjusted hazards ratios (aHRs) with their corresponding 95% confidence interval and *p* value. In all the above analyses, a *p* value < 0.05 was considered statistically significant. Statistical analyses were performed using the Statistical Package for the Social Sciences version 26 (SPSS, Armonk, NY, USA, IBM Corp.).

### 2.5. Ethics Approval

We obtained ethical approval from the institutional review board (National Healthcare Group Domain Specific Review Board, Reference Number: 2021/00623).

## 3. Results

### 3.1. Demographics

**Figure 1:**
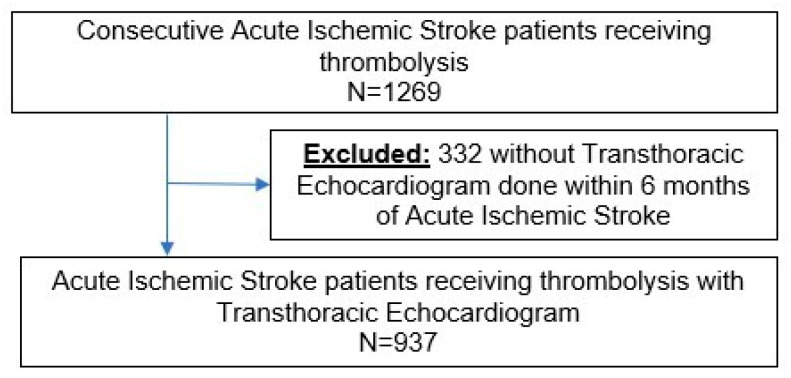
Flowchart of patient selection.

Of the 1269 consecutive AIS patients who received IV thrombolysis between January 2006 and December 2018, a total of 937 patients with AIS who underwent IV thrombolysis (Figure 1) were included in this study (Table 1). The mean duration of follow-up was 5.84 ± 3.69 years. LVSD was observed in 190 (25.4%) patients. There were fewer females in patients with LVSD (30.1% vs. 40.4%, *p* = 0.010). Dyslipidemia (62.1% vs. 49.9%, *p* = 0.003), diabetes mellitus (52.6% vs. 37.5%, *p* < 0.001), atrial fibrillation (36.3% vs. 28.4%, *p* = 0.033), ischemic heart disease (68.4% vs. 19.4%, *p* < 0.001), HF (78.9% vs. 6.2%, *p* < 0.001), previous percutaneous coronary intervention (18.4% vs. 5.6%, *p* < 0.001) and previous coronary artery bypass graft (11.1% vs. 2.8%, *p* < 0.001) were more common in patients with LVSD. Baseline NIHSS on arrival was notably higher in patients with LVSD (18.0 vs. 15.0, *p* = 0.003).

Stroke characteristics are highlighted in Table 2. Time of stroke onset to needle (157.3 ± 57.8 min vs. 165.0 ± 62.4 min, *p* = 0.133) and door-to-needle time (82.7 ± 41.3 min vs. 81.9 ± 50.5 min, *p* = 0.856) were similar in AIS patients with and without LVSD. The type of ischemic stroke based on the TOAST classification (*p* < 0.001) and success of recanalization post IV thrombolysis (*p* = 0.006) were noted to be significant between both groups of patients. Large-artery atherosclerosis and cardio-embolism accounted for the AIS etiology in most patients with and without LVSD (60.0% vs. 54.9%).

However, in terms of stroke outcomes (Table 3), fewer patients with LVSD achieved functional independence at 3 months (45.3% vs. 55.0%, *p* = 0.016). Functional independence rates were similar whether the patient had a subsequent stroke/TIA. A significantly greater proportion of AIS patients with LVSD met with outcomes of all-cause mortality (58.9% vs. 29.6%, *p* < 0.001), subsequent HF admission (28.4% vs. 6.6%, *p* < 0.001) and subsequent MI (16.3% vs. 6.2%, *p* < 0.001), but the rates of symptomatic ICH (5.3% vs. 3.1%, *p* = 0.145) and recurrent stroke/TIA (15.3% vs. 17.0%, *p* = 0.566) were similar between both arms.

### 3.2. mRS Outcomes at 3 Months

**Figure 2:**
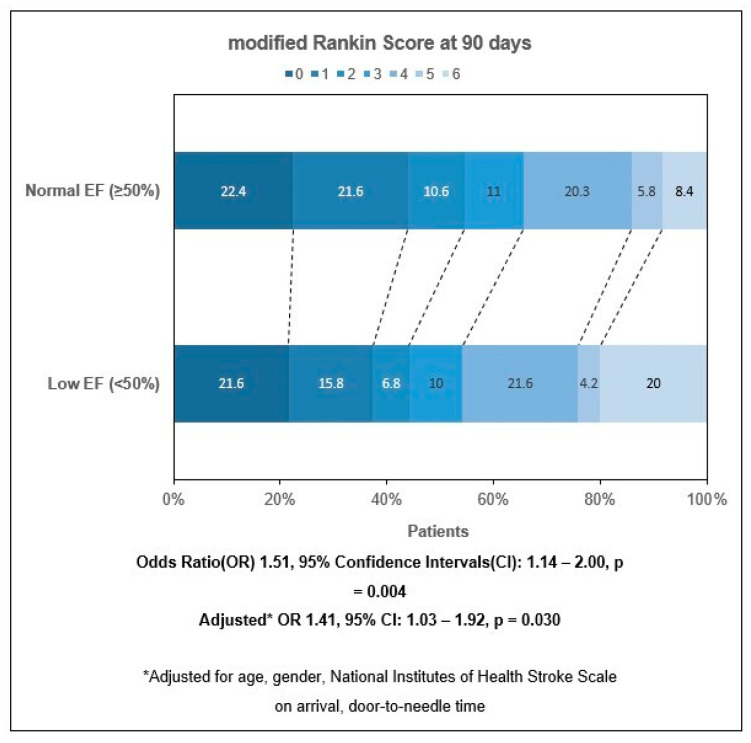
Univariate and multivariate Ordinal Regression for modified Rankin Scale at 3 months. Abbreviations: CI, Confidence Intervals; EF, Ejection Fraction; OR, Odds Ratio.

On ordinal shift regression analysis, LVSD (OR 1.51, 95% CI 1.14 to 2.00; *p* = 0.004) was associated with an unfavorable shift in mRS outcomes at 3 months. LVSD remained significant even after adjusting for age, sex, NIHSS on arrival and door-to-needle time (aOR 1.41, 95% CI 1.03 to 1.92; *p* = 0.030) (Figure 2). Among patients with and without LVSD, functional independence was achieved in 44.2% and 54.6% of patients, respectively, at 3 months.

### 3.3. Outcomes of All Cause Mortality, Subsequent HF Admission and Subsequent MI Event

On multivariate Cox regression analysis, LVSD was an independent predictor for all-cause mortality (aHR 3.38, 95% CI 1.74 to 6.54; *p* < 0.001), subsequent HF admission (aHR 4.23, 95% CI 2.17 to 8.26; *p* < 0.001) and subsequent MI event (aHR 2.49, 95% CI 1.44 to 4.32; *p* = 0.001) (Table 4, Table 5 and Table 6) after accounting for clinically relevant demographic variables such as age, sex and race; cardiovascular comorbidities such as hypertension, dyslipidemia, diabetes mellitus, atrial fibrillation and ischemic heart disease; and ischemic stroke parameters such as TOAST score, NIHSS score on arrival and post thrombolysis recanalization success. For all-cause mortality, age (aHR 1.07, 95% CI 1.04 to 1.09; *p* < 0.001), presence of diabetes mellitus (aHR 1.84, 95% CI 1.10 to 3.07; *p* = 0.020) and atrial fibrillation (aHR 0.327, 95% CI 0.115 to 0.930; *p* < 0.036) were also noted to be significant factors. In the regression analysis of subsequent heart failure admission, age (aHR 1.02, 95% CI 0.995 to 1.04; *p* = 0.008) was also identified to be a significant factor, while in the analysis for subsequent MI, diabetes mellitus remained significant (aHR 2.53, 95% CI 1.53 to 4.19; *p* < 0.001). However, LVSD was not a significant predictor of increased recurrent stroke/TIA events (aHR 1.15, 95% CI 0.77 to 1.72; *p* = 0.496) and remained insignificant after accounting for AF and IHD (aHR 1.19, 95% CI 0.76 to 1.86; *p* = 0.461).

## 4. Discussion

Among AIS patients who underwent IV thrombolysis, the presence of LVSD was associated with higher rates of all-cause mortality, subsequent HF admissions, MI events, as well as poorer functional outcomes at 3 months, even after adjusting for age and other comorbidities. LVSD was not significantly associated with recurrent stroke/TIA events.

IV thrombolysis remains the standard of care for AIS patients worldwide [29,30,31]. Favorable outcomes of improved functional outcomes and reduced mortality have been observed when thrombolytic therapy is given up to 4.5 h from symptom onset [32]. Despite close associations between cardiac disease and AIS [3], most stroke registries and contemporary thrombolytic trials [33,34,35,36] tend not to report baseline data on cardiac diseases.

In this present cohort study of AIS patients who underwent IV thrombolysis, LVSD was associated with increasingly worse mRS outcomes at 3 months, even after adjusting for the difference in NIHSS scores on arrival between the two groups of patients. Our results are consistent with several studies that have similar baseline LVEF characteristics and report unfavorable functional outcomes in AIS patients at 3 months when treated with IV thrombolysis or ET [13,15,37]. Few studies looked at mRS outcomes in a subgroup of AIS patients that specifically underwent IV thrombolysis. One could posit that LVSD has a bidirectional impact on both the brain and the heart. While IV thrombolysis serves to improve cerebral reperfusion, our study findings instead support the theory that poor LVEF may still contribute to poor cerebral perfusion [16,38], which is possibly attributable to decreased stroke volume and decreased autoregulation function of the brain, changing brain structure that was not manifested prior to stroke onset [39]. Moreover, other possible mechanisms include neurohormonal factors [6] acting on cardiac cells in LVSD patients, which could impair cardiomyocytes and reduce effort tolerance during rehabilitation, thereby hindering [40] the neurorehabilitation process, which is often complicated by cardiac arrhythmia and physical impairments [37], hence negatively impacting mRS outcomes. While more research is required to establish the underlying mechanism, our findings remain clinically relevant wherein reducing complications of LVEF can possibly improve mRS outcomes, paving the way for prophylactic therapy.

To the best of our knowledge, while other studies have shown increased cardiac morbidity and mortality after AIS [41], these studies typically examine a heterogenous group of AIS patients and have not specifically analyzed patients undergoing IV thrombolysis. Furthermore, in many studies, the prevalence of LVSD in AIS patients is often not reported [12,13,14,15,42,43,44]. Hence, not only does this study include baseline cardiac parameters that provide further context to this cohort of AIS patients, this study also adds on to the prevailing literature by showing an association between LVSD and increased risk of subsequent cardiac events in AIS patients. Current research strongly supports the theory where stress responses induced by AIS cause over activation of central autonomic neural networks, resulting in dysfunction involving the autonomic nervous system [45]. These supporting theories also suggest that the underlying mechanisms for brain–heart interactions include the activation of the hypothalamic-pituitary-adrenal axis, catecholamine surge and sympathetic and parasympathetic regulation along with immune and inflammation responses, which causes cardiac injury post stroke [46]. Our findings are clinically relevant: currently, echocardiography is highly recommended in the evaluation of stroke and, hence, this can allow us to better prognosticate the clinical recovery of AIS patients [47]. Through evaluating LVEF, LVSD in AIS patients identified to be of increased risk of adverse clinical outcomes can also benefit from greater resources allocated to focus on closer monitoring and more intensive post-stroke rehabilitation.

Additionally, most studies do not look at recurrent stroke/TIA events; our study revealed LVSD to have no association in AIS patients, even after accounting for AF and IHD. While pre-stroke HF remains closely associated with more severe strokes, possibly due to cardiac embolism causing greater infarction [6], another study also concurred that the association of recurrent stroke with poor LVEF has low statistical power [48]. A lack of sufficient relevant studies suggest that this association remains unclear, although some studies highlight other significant predictors to include size or diameter of the left atrium [49] and atrial fibrillation [50,51]. Other studies also raise the possibility that LVSD based on echocardiography might not be the best surrogate marker in determining cardiac function to determine recurrent stroke, with several papers proposing other echocardiography markers of cardiac function and structure as risk factors [48]. Further studies are required to compare echocardiography markers as predictors for recurrent stroke.

Surprisingly, apart from LVSD, only the presence of diabetes mellitus remained a significant factor associated with negative outcomes in our study. This is noteworthy, as diabetes mellitus has been associated with worse post-stroke recovery after rehabilitation [52], and this negatively predicts functional outcomes in AIS patients [53]. While Tokgoz et al. [54] reported the converse—a history of diabetes mellitus is not significant in determining mortality in AIS patients, the study also similarly details median glucose levels that are significantly higher in AIS patients with mortality. One possible theory suggested that tissue acidosis as a result of anaerobic glycolysis and free radical production leads to the disruption of the blood–brain barrier, resulting in cerebral edema, hence increasing the risk of hemorrhagic transformation [55] and leading to a poorer prognosis, making the optimization of glycemic status pertinent.

Interestingly, the prevalence of hypertension, on the other hand, was not increased in patients with LVSD. Many trials have demonstrated a strong association between hypertension and LVSD [56], suggesting underlying mechanisms to include changes in cardiac structure and function by increasing the left ventricular afterload and peripheral vascular resistance, resulting in cardiac remodeling and, hence, LVSD [57]. Many comorbidities could lead to LVSD, and hypertension is a known factor. In our study, we were limited by the comprehensiveness of the patient’s comorbidity history, including compliance with hypertensive treatment and the duration of hypertension in our patient population. In most of the above studies [56], longstanding and chronic hypertension allowed for structural cardiac changes. Postulated explanations in our study regarding this difference in results can be attributed to the characteristics of our population studied, which might possibly have good adherence to anti-hypertensive treatment or hypertension that is not longstanding. These can further explain why hypertension was not a significant factor between patients with LVSD and normal LVEF in our study.

### 4.1. Moving Forward

In the preventive treatment of LVSD, especially in AIS patients with strong cardiovascular risk factors such as AF and IHD, newer studies support the use of combination antiplatelet and anticoagulant therapy in cardiovascular prevention [58,59], with some stressing the importance of antiplatelet therapy [60] and even antithrombotic therapy [61]. Admittedly, this might further increase the risk of complications, such as heightened bleeding risk, especially in the AIS patient population undergoing IV thrombolysis [62]. Based on our knowledge, combination therapy is largely given in specific contexts such as in patients undergoing PCI or with atherosclerotic cardiovascular disease as per the guidelines by the American College of Cardiology [63]. Hence, as this study seeks to identify an association between LVSD and AIS outcomes in patients undergoing IV thrombolysis, we were limited by details on the medication history of our population. This study therefore paves the way for future research to better underscore the impact of cardiovascular prevention medication with AIS patients’ outcomes.

The role of antithrombotic agents in the prevention of stroke in patients specifically with HF has been much less investigated. The Warfarin versus Aspirin in Reduced Cardiac Ejection Fraction (WARCEF) Study Group [64] compared double-blinded warfarin to aspirin. In this trial, while the stroke rate was reduced from 1.36 per 100 patient-years on aspirin to 0.72 per 100 patient-years on warfarin, the rate of general major bleeding increased from 2.7% on aspirin to 5.8% on warfarin. The trial concluded that the choice between warfarin and aspirin should be individualized, as there was no significant overall difference in the primary outcome between treatment with warfarin and treatment with aspirin [65]. In fact, the reduced risk of ischemic stroke with warfarin was offset by an increased risk of major hemorrhage. Similarly, in the COMMANDER HF trial (A Study to Assess the Effectiveness and Safety of Rivaroxaban in Reducing the Risk of Death, Myocardial Infarction, or Stroke in Participants with Heart Failure and Coronary Artery Disease Following an Episode of Decompensated Heart Failure) [66], rivaroxaban was not associated with a significantly lower rate of stroke recurrence in patients with HF. The similarities in results between the WARCEF and COMMANDER HF trial could possibly be due to the diverse underlying causes of HF. Several new meta-analyses [67] have also supported the use of antiplatelet therapy in the secondary prevention of stroke in AIS patients with vascular risk factors, which could potentially be useful. Therefore, as each underlying HF etiology for every patient differs, careful elucidation through a thorough work up on the cause of LVSD will remain crucial for personalized and effective treatment [67,68] in the preventive role of antithrombotic therapy in HF patients with stroke. Nonetheless, further research in this aspect would be required regarding the use of antithrombotic agents in stroke patients with HF.

Recent advancements in the understanding of the stroke-heart syndrome have further illustrated the intricate relationship between LVSD and AIS, stressing the importance of LVSD optimization [69]. Currently, referencing the European Society of Cardiology, pharmacotherapy for the management of HF includes the use of beta-blockers, angiotensin-converting enzyme inhibitors, angiotensin-receptor blocker or angiotensin receptor-neprilysin inhibitors, mineralocorticoid receptor antagonists and sodium glucose co-transporter 2 inhibitors [70]. However, one of the considerations in the management of AIS patients is the need for permissive HTN. With a blood pressure goal of ≤220/120 mmHg for the first 24 to 48 h if the patient is not undergoing any acute intervention such as IV thrombolysis or endovascular thrombectomy as per the current guidelines of American Heart Association/American Stroke Association [71], the introduction of the recommended HF medications may further complicate the management of AIS due to possible side effects on the reduction in blood pressure. While our study has highlighted the impact and association of LVSD with clinical outcomes in AIS patients post IV thrombolysis, future research should also investigate strategies to optimize HF therapeutics in AIS patients with LVSD.

### 4.2. Strengths and Limitations

Several limitations should be highlighted when interpreting the study results. Firstly, as our study is a retrospective study, we could only show associations but not causation. As this is also a single-center study, results need to be validated in other cohorts. Secondly, recruitment was conducted over an extended time which spanned across pre- and post-thrombectomy.

Lead time bias may be possible. Furthermore, we could only track outcomes of patients that presented to our institution, and hence there may have been loss-to-follow-up bias if patients subsequently presented to other centers for treatment. Thirdly, as LVSD in this study was taken to be LVEF < 50%, other baseline patient information that was unavailable, including further details of patients’ comorbidities, subtype of stroke etiology and cardiac functional status such as New York Heart Association class, NT Pro-BNP biomarker and baseline medications, would have been useful to fully evaluate whether HF contributed to poorer functional outcomes. Thus, whether current findings may be generalized to all HF patients remains to be clarified.

As with most observation studies, uncontrolled confounding factors were present in this study; nonetheless, this study has a relatively large cohort size with data on LVEF and the results still provide greater insights into the impact of LVSD on both cardiovascular and neurological functional outcomes in AIS patients undergoing IV thrombolysis.

## 5. Conclusions

In conclusion, we demonstrated the association of LVSD with outcomes in AIS patients undergoing IV thrombolysis. LVSD was associated with poorer functional outcomes as well as worse mRS outcomes at 3 months, even after adjusting for age and cardiovascular comorbidities. LVSD was also associated with higher rates of all-cause mortality, subsequent HF admissions and MI events.

## Figures and Tables

**Table 1:** Demographics of Acute Ischemic Stroke patients with Normal Left Ventricular Ejection Fraction and Left Ventricular Systolic Dysfunction.

Variables	LVSD (*n* = 190)	Normal LVEF (*n* = 747)	*p*-Value
**Baseline characteristics**			
Age (years), mean (SD)	65.4 (13.8)	65.2 (13.5)	0.877
Female, % (*n*)	30.1 (56)	40.4 (296)	**0.010**
Ethnicity, % (*n*)			**0.002**
Chinese	56.3 (94)	69.5 (459)	**0.001**
Malay	30.5 (51)	18.9 (125)	**0.001**
Indian	9.0 (15)	5.9 (39)	0.151
Others	4.2 (7)	5.6 (37)	0.467
**Comorbidities**			
Hypertension, % (*n*)	76.3 (145)	77.1 (576)	0.817
Dyslipidemia, % (*n*)	62.1 (118)	49.9 (373)	**0.003**
Diabetes Mellitus, % (*n*)	52.6 (100)	37.5 (280)	**<0.001**
Atrial Fibrillation, % (*n*)	36.3 (69)	28.4 (212)	**0.033**
Ischemic Heart Disease, % (*n*)	68.4 (130)	19.4 (145)	**<0.001**
Heart Failure, % (*n*)	78.9 (150)	6.2 (46)	**<0.001**
Previous Percutaneous Coronary Intervention, % (*n*)	18.4 (35)	5.6 (42)	**<0.001**
Previous Coronary Artery Bypass Graft, % (*n*)	11.1 (21)	2.8 (21)	**<0.001**
National Institutes of Health Stroke Scale on arrival, median (IQR)	18.0 (11.0–22.0)	15.0 (8.0–21.0)	**0.003**

Bold values are statistically significant. Abbreviations: CI, Confidence Intervals; IQR, Inter-quartile range; LVEF, Left Ventricular Ejection Fraction (LVEF); LVSD, Left Ventricular Systolic Dysfunction; SD, Standard Deviation.

**Table 2:** Stroke Characteristics of Acute Ischemic Stroke patients with Normal Left Ventricular Ejection Fraction and Left Ventricular Systolic Dysfunction.

Variables	LVSD (*n* = 190)	Normal LVEF (*n* = 747)	*p*-Value
**Investigation findings**			
Left Ventricular Ejection Fraction (%), mean (SD)	33.5 (9.84)	63.0 (5.02)	**<0.001**
Site of occlusion			0.179
M1—Middle Cerebral Artery, % (*n*)	34.2 (65)	29.2 (218)	0.178
M2—Middle Cerebral Artery, % (*n*)	2.6 (5)	1.1 (8)	0.154
Terminal Internal Carotid Artery, % (*n*)	14.2 (27)	11.8 (88)	0.362
No Large Vessel Occlusion, % (*n*)	13.7 (26)	27.7 (207)	**<0.001**
Basilar Artery, % (*n*)	6.3 (12)	7.5 (56)	0.575
Tandem, % (*n*)	7.9 (15)	8.4 (63)	0.810
TOAST Classification			**<0.001**
1	17.4 (33)	23.2 (173)	
2	42.6 (81)	31.7 (237)	
3	3.2 (6)	9.1 (68)	
4	0 (0)	1.9 (14)	
5	18.4 (35)	12.2 (91)	
**Procedure**			
Time from stroke onset to needle (min), mean (SD)	157.3 (57.8)	165.0 (62.4)	0.133
Time from door to needle (min), mean (SD)	82.7 (41.3)	81.9 (50.5)	0.856
Recanalization of vascular occlusion, % (*n*)			**0.006**
Complete recanalization, % (*n*)	20.5 (39)	21.2 (158)	
Partial recanalization, % (*n*)	7.4 (14)	6.3 (47)	
No recanalization, % (*n*)	18.4 (35)	13.1 (98)	
No occlusion, % (*n*)	13.7 (26)	25.6 (191)	

Bold values are statistically significant. Abbreviations: CI, Confidence Intervals; IQR, Inter-quartile range; LVEF, Left Ventricular Ejection Fraction (LVEF); LVSD, Left Ventricular Systolic Dysfunction; SD, Standard Deviation.

**Table 3:** Outcomes of Acute Ischemic Stroke patients with Normal Left Ventricular Ejection Fraction and Left Ventricular Systolic Dysfunction.

Variables	LVSD (*n* = 190)	Normal LVEF (*n* = 747)	*p*-Value
**Outcomes**			
Modified Rankin Scale (mRS) at 3 months, % (*n*)			**<0.001**
0	21.6 (41)	22.4 (167)	0.818
1	15.8 (30)	21.6 (161)	0.078
2	6.8 (13)	10.6 (79)	0.123
3	10.0 (19)	11.0 (82)	0.698
4	21.6 (41)	20.3 (152)	0.708
5	4.2 (8)	5.8 (43)	0.402
6	20.0 (38)	8.4 (63)	**<0.001**
Functional independence at 3 months (mRS 0–2), % (*n*)	45.3 (86)	55.0 (411)	**0.016**
All-cause mortality, % (*n*)	58.9 (112)	29.6 (221)	**<0.001**
Subsequent heart failure admission, % (*n*)	28.4 (54)	6.6 (49)	**<0.001**
Subsequent myocardial infarction, % (*n*)	16.3 (31)	6.2 (4.6)	**<0.001**
Recurrent stroke/transient ischemic attack, % (*n*)	15.3 (29)	17.0 (127)	0.566
Symptomatic intracerebral hemorrhage, % (*n*)	5.3 (10)	3.1 (23)	0.145

Bold values are statistically significant. Abbreviations: CI, Confidence Intervals; IQR, Inter-quartile range; LVEF, Left Ventricular Ejection Fraction (LVEF); LVSD, Left Ventricular Systolic Dysfunction; SD, Standard Deviation.

**Table 4:** Multivariate Cox regression for all-cause mortality.

	Univariate Analysis		Multivariate Analysis	
Covariates	Hazards Ratio (95% CI)	*p* Value	Adjusted Hazards Ratio (95% CI)	*p* Value
Left Ventricular Systolic Dysfunction	0.399 [0.318, 0.501]	**<0.001**	**3.38 [1.74, 6.54]**	**<0.001**
Age	1.04 [1.03, 1.05]	**<0.001**	**1.07 [1.04, 1.09]**	**<0.001**
Female Sex	1.01 [0.809, 1.26]	0.921		
Race				
Chinese (REF)				0.441
Malay	1.29 [0.989, 1.69]	0.061	1.25 [0.676, 2.30]	0.479
Indian	0.952 [0.587, 1.54]	0.841	1.90 [0.719, 5.00]	0.196
Other	0.434 [0.204, 0.922]	**0.030**	0.615 [0.159, 2.38]	0.481
Hypertension	1.26 [0.963, 1.64]	0.093		
Dyslipidemia	1.29 [1.04, 1.61]	**0.022**	0.914 [0.545, 1.53]	0.733
Diabetes Mellitus	1.72 [1.38, 213]	**<0.001**	1.84 [1.10, 3.07]	**0.020**
Atrial Fibrillation	1.39 [1.11, 1.74]	**0.004**	0.327 [0.115, 0.930]	**0.036**
Ischemic Heart Disease	2.04 [1.64, 2.54]	**<0.001**	1.63 [0.895, 2.96]	0.111
National Institutes of Health Stroke Scale on arrival	1.06 [1.04, 1.07]	**<0.001**	1.03 [0.991, 1.07]	0.129
TOAST Classification				
1 (REF)		**0.001**		0.054
2	1.57 [1.10, 2.26]	**0.014**	1.83 [0.627, 5.33]	0.269
3	0.704 [0.391, 1.27]	0.242	0.814 [0.288, 2.30]	0.699
4	0.761 [0.230, 2.51]	0.653	0.978 [0.082, 11.7]	0.986
5	0.703 [0.432, 1.14]	0.156	0.351 [0.151, 0.817]	**0.015**
Recanalization				
No occlusion (REF)		**0.002**		0.389
Complete recanalization	2.02 [1.33, 3.07]	**0.001**	1.33 [0.598, 2.94]	0.482
Partial recanalization	1.37 [0.732, 2.55]	**0.327**	0.931 [0.327, 2.65]	0.893
No recanalization	2.13 [1.34, 3.38]	**0.001**	1.84 [0.764, 4.41]	0.175

Bold values are statistically significant. Abbreviations: CI, Confidence Intervals.

**Table 5:** Multivariate Cox regression for subsequent heart failure admission.

	Univariate Analysis		Multivariate Analysis	
Covariates	Hazards Ratio (95% CI)	*p* Value	Adjusted Hazards Ratio (95% CI)	*p* Value
Left Ventricular Systolic Dysfunction	6.75 [4.57, 9.95]	**<0.002**	4.23 [2.17, 8.26]	**<0.001**
Age	1.03 [1.01, 1.04]	**0.001**	1.02 [0.995, 1.04]	**0.008**
Female Sex	0.895 [0.598, 1.34]	0.590		
Race				
Chinese (REF)		0.752		
Malay	1.24 [0.763, 2.03]	0.382		
Indian	1.31 [0.627, 2.75]	0.472		
Other	1.25 [0.540, 2.89]	0.607		
Hypertension	1.05 [0.665, 1.64]	0.848		
Dyslipidemia	1.85 [1.23, 2.79]	**0.003**	1.50 [0.833, 2.72]	0.175
Diabetes Mellitus	2.14 [1.45, 3.16]	**<0.001**	0.966 [0.538, 1.73]	0.907
Atrial Fibrillation	1.94 [1.31, 2.86]	**0.001**	1.54 [0.835, 2.83]	0.168
Ischemic Heart Disease	3.21 [2.18, 4.72]	**<0.001**	1.09 [0.566, 2.10]	0.797
National Institutes of Health Stroke Scale on arrival	0.992 [0.965, 1.02]	0.552		
TOAST Classification				
1 (REF)		0.329		
2	1.61 [0.925, 2.81]	0.092		
3	0.821 [0.316, 2.13]	0.684		
4	0.715 [0.089, 5.76]	0.753		
5	1.26 [0.618, 2.56]	0.528		
Recanalization				
No occlusion (REF)		**0.032**		0.071
Complete recanalization	2.35 [1.24, 4.44]	**0.009**	1.81 [0.904, 3.63]	0.094
Partial recanalization	1.12 [0.394, 3.20]	0.830	0.727 [0.235, 2.25]	0.581
No recanalization	1.13 [0.509, 2.52]	0.760	0.775 [0.330, 1.83]	0.560

Bold values are statistically significant. Abbreviations: CI, Confidence Intervals.

**Table 6:** Multivariate Cox regression for subsequent myocardial infarction.

	Univariate Analysis		Multivariate Analysis	
Covariates	Hazards Ratio (95% CI)	*p* Value	Adjusted Hazards Ratio (95% CI)	*p* Value
Left Ventricular Systolic Dysfunction	3.56 [2.24, 5.66]	**<0.001**	2.49 [1.44, 4.32]	**0.001**
Age	1.02 [1.00, 1.04]	**0.034**	1.01 [0.994, 1.03]	0.187
Female Sex	1.08 [0.677, 1.73]	0.742		
Race				
Chinese (REF)		0.799		
Malay	1.15 [0.639, 2.07]	0.642		
Indian	0.963 [0.345, 2.69]	0.943		
Other	0.548 [0.133, 2.26]	0.406		
Hypertension	1.27 [0.721, 2.24]	0.405		
Dyslipidemia	2.29 [1.39, 3.76]	**0.001**	1.41 [0.819, 2.44]	0.214
Diabetes Mellitus	3.18 [1.98, 5.11]	**<0.001**	2.53 [1.53, 4.19]	**<0.001**
Atrial Fibrillation	1.86 [1.18, 2.95]	**0.008**	1.55 [0.960, 2.51]	0.076
Ischemic Heart Disease	2.61 [1.66, 4.11]	**<0.001**	1.35 [0.786, 2.31]	0.278
National Institutes of Health Stroke Scale on arrival	1.00 [0.969, 1.03]	0.995		
TOAST Classification				
1 (REF)		0.510		
2	1.48 [0.653, 2.13]	0.586		
3	0.562 [0.185, 1.71]	0.311		
4	0.00 [0.00, 0.00]	0.999		
5	0.667 [0.283, 1.57]	0.355		
Recanalization				
No occlusion (REF)		0.895		
Complete recanalization	0.754 [0.370, 1.54]	0.436		
Partial recanalization	0.879 [0.316, 2.45]	0.806		
No recanalization	0.888 [0.411, 1.92]	0.763		

Bold values are statistically significant. Abbreviations: CI, Confidence Intervals.

## Data Availability

Data are unavailable due to privacy or ethical restrictions.
